# Development of acetabular retroversion in LCPD hips—an observational radiographic study from early stage to healing

**DOI:** 10.1007/s00402-022-04612-0

**Published:** 2022-10-23

**Authors:** Christiane Sylvia Leibold, Patrick Whitlock, Florian Schmaranzer, Kai Ziebarth, Moritz Tannast, Simon Damian Steppacher

**Affiliations:** 1 Department of Orthopaedic Surgery and Traumatology, Inselspital, Bern University Hospital, University of Bern, Freiburgstrasse, 3010 Bern, Switzerland; 2 Division of Orthopaedic Surgery, Cincinnati Children’s Hospital Medical Center, Cincinnati, OH USA; 3 Department of Diagnostic-, Interventional- and Paediatric Radiology, Inselspital, Bern University Hospital, University of Bern, Freiburgstrasse, 3010 Bern, Switzerland; 4 Department of Pediatric Surgery, Inselspital, Bern University Hospital, University of Bern, Freiburgstrasse, 3010 Bern, Switzerland; 5 Department of Orthopaedic Surgery and Traumatology, HFR Cantonal Hospital, University of Fribourg, Chemin des Pensionnats 2-6, Villars-sur-Glâne, 1752 Fribourg, Switzerland

**Keywords:** Legg–Calvé–Perthes Disease, Acetabular retroversion, Morbus perthes, LCPD, Femoroacetabular impingement

## Abstract

**Background:**

Acetabular retroversion is observed frequently in healed Legg–Calvé–Perthes disease (LCPD). Currently, it is unknown at which stage and with what prevalence retroversion occurs because in non-ossified hips, retroversion cannot be measured with standard radiographic parameters.

**Methods:**

In a retrospective, observational study; we examined pelvic radiographs in children with LCPD the time point of occurrence of acetabular retroversion and calculated predictive factors for retroversion. Between 2004 and 2017, we included 55 children with a mean age of 5.7 ± 2.4 years at diagnosis. The mean radiographic follow-up was 7.0 ± 4.4 years. We used two new radiographic parameters which allow assessment of acetabular version in non-ossified hips: the pelvic width index and the ilioischial angle. They are based on the fact that the pelvic morphology differs depending on the acetabular version. These parameters were compared among the four Waldenström stages and to the contralateral side. Logistic regression analysis was performed to determine predictive factors for acetabular retroversion.

**Results:**

Both parameters differed significantly among the stages of Waldenström (*p* < 0.003 und 0.038, respectively). A more retroverted acetabulum was found in stage II and III (prevalence ranging from 54 to 56%) compared to stage I and IV (prevalence ranging from 23 to 39%). In hips of the contralateral side without LCPD, the prevalence of acetabular retroversion was 0% in all stages for both parameters. Predictive factors for retroversion were younger age at stage II and IV, collapse of the lateral pillar in stage II or a non-dysplastic hip.

**Conclusions:**

This is the first study evaluating acetabular version in children with LCPD from early stage to healing. In the developing hip, LCPD may result in acetabular retroversion and is most prevalent in the fragmentation (stage II) and early healing stage (stage III). Partial correction of acetabular retroversion can occur after healing. This has a potential clinical impact on the timing and type of surgical correction, especially in pelvic osteotomies for correction of acetabular version.

**Level of evidence:**

Level III, retrospective observational study.

## Introduction

Legg–Calvé–Perthes Disease (LCPD) affects the developing femoral head and may result in retroversion of the acetabulum. Among different hip disorders, the prevalence of acetabular retroversion is reported highest in LCPD ranging from 31 to 49% [5]. Since LCPD typically occurs before skeletal maturity (typical onset from 4 to 8 years [2]), the shape of the acetabulum cannot be accurately assessed on radiographs due to the pending ossification. Most publications on acetabular version in LCPD are reported in skeletally mature hips with healed LCPD [4, 11, 12]. Results on acetabular version in the early stage of LCPD are either performed using CT or MRI and have no evaluation over time [12, 17, 26].

Acetabular retroversion is not an isolated deformity of the acetabulum but rather the entire hemipelvis [1, 6, 8, 10, 19, 22]. New and indirect radiographic parameters have been described to evaluate acetabular retroversion, based on the morphology of the hemipelvis rather than the bony morphology of the acetabulum itself [22]. Using these indirect radiographic parameters allows evaluation of acetabular version before ossification of the acetabulum. Therefore, we asked: (1) In what stage according to Waldenström [24] does acetabular retroversion occur over the course of LCPD? (2) Does acetabular version of the contralateral hip in children with unilateral LCPD change as well? And (3) are there any factors predicting acetabular retroversion in stage II (fragmentation) or IV (healed) of LCPD according to Waldenström?

## Patients and methods

**Fig. 1:**
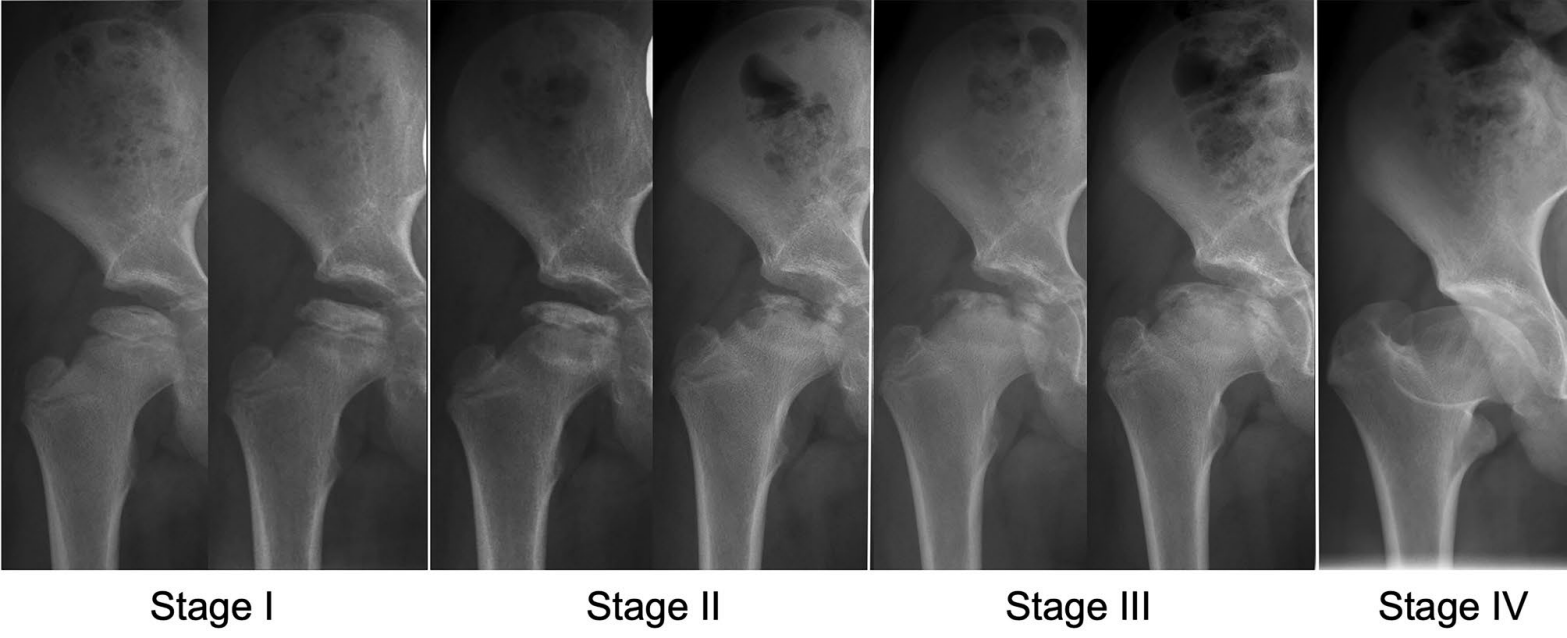
Natural course of Legg–Calvé–Perthes Disease (LCPD) staged according to Waldenström classification [24] into four stages: Stage I with sclerosis or loss of height of the epiphysis; Stage II with the beginning of fracturing or advanced fragmentation of the epiphysis; Stage III with early new bone formation; and Stage IV with complete healing of the epiphysis

We performed a retrospective, observational study to evaluate acetabular version in children diagnosed with LCPD from the early stage of the disease to healing. Between June 2004 and October 2017, 98 children (101 hips) were diagnosed with LCPD. Thereof, we excluded 43 children (44%; 46 hips) with radiographs without correct centering or malorientation of the pelvis, with radiographs not including a minimum of three out of four stages according to Waldenström (Fig. 1) or children with bilateral LCPD. This resulted in 55 children (55 hips) with LCPD (Table 1). The mean age at diagnosis of LCPD was 5.7 ± 2.4 (2–13) years and a majority of 40 children were male children (78%). All human and animal studies have been approved by the appropriate ethics committee and have been performed in accordance with the ethical standards of the 1964 Declaration of Helsinki and its later amendments.

**Table 1:** Demographic and radiographic information of the patient series with Legg–Calvé–Perthes disease

Parameter	Value
Total number of hips (patients)	55 (55)
Age at diagnosis (years)	5.7 ± 2.4 (2.0–13.0)
Male (number of patients [% of all patients])	40 (73)
Number of hips in each Waldenström stage (number of hips [% of all hips])	
Stage I	41 (75)
Stage II	54 (98)
Stage III	52 (95)
Stage IV	43 (78)
Mean age in each Waldenström stage	
Stage I	6.1 ± 2.5 (2.7–13.0)
Stage II	6.2 ± 2.3 (2.0–13.8)
Stage III	7.3 ± 2.4 (3.1–15.6)
Stage IV	9.3 ± 2.4 (5.7–16.2)
Lateral pillar classification* (hips [% of all hips])	
Group a	9 (17)
Group b	32 (59)
Group c	13 (24)
Stulberg classification^†^ (hips [% of all hips])	
Stage 1	6 (13)
Stage 2	12 (28)
Stage 3	12 (28)
Stage 4	11 (26)
Stage 5	2 (5)
Positive ischial spine sign^†^ (hips [% of all hips])	
LCPD	16 (37)
Contralateral side without LCPD	0 (0)
Positive cross-over sign^†^ (hips [% of all hips])	
LCPD	17 (39)
Contralateral side without LCPD	0 (0)
Retroversion Index > 30%^†^ (hips [% of all hips])	
LCPD	13 (30)
Contralateral side without LCPD	0 (0)
Positive posterior wall sign^†^ (hips [% of all hips])	
LCPD	16 (37)
Contralateral side without LCPD	0 (0)

Continuous parameters are expressed as mean ± standard deviation and range in parenthesis

*LCPD* Legg–Calvé–Perthes disease

*classified in the 54 hips with LCPD in Waldenström stage II; ^†^classified in the 43 hips with LCPD in Waldenström stage IV

**Fig. 2:**
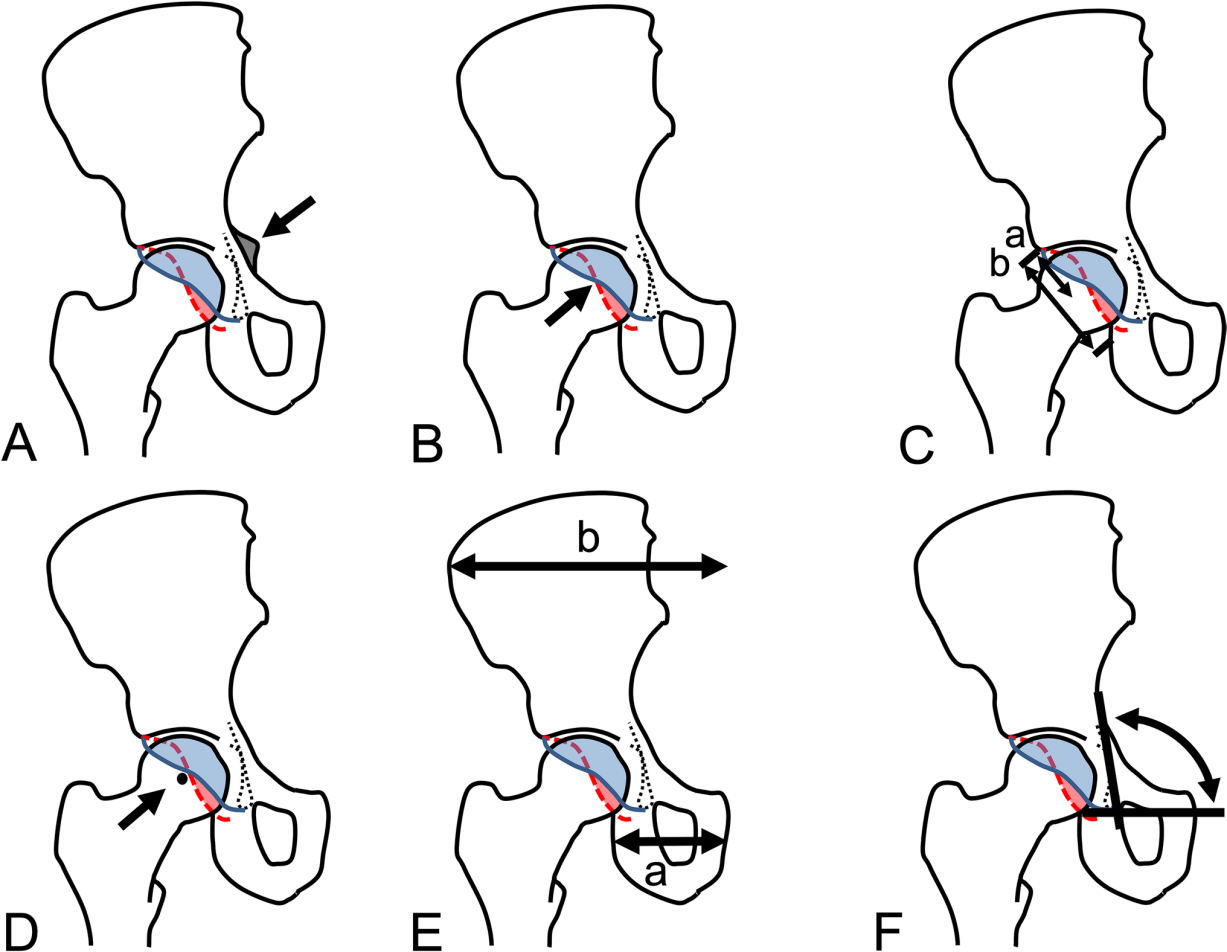
Radiographic parameters to evaluate acetabular version (See definitions of these parameters in Table 2.). Standard radiographic parameters to evaluate acetabular retroversion require an ossified acetabulum and include **A** the ischial spine sign [10], **B** cross-over sign [8, 16], **C** the retroversion index [16, 23], and **D** the posterior wall index [16, 23]. Indirect parameters allow to assess acetabular version in both hips with and without complete ossification of the acetabulum evaluating the shape of the hemipelvis. These parameters include **E** the pelvic width index [22] and **F** the ilioischial angle

**Fig. 3:**
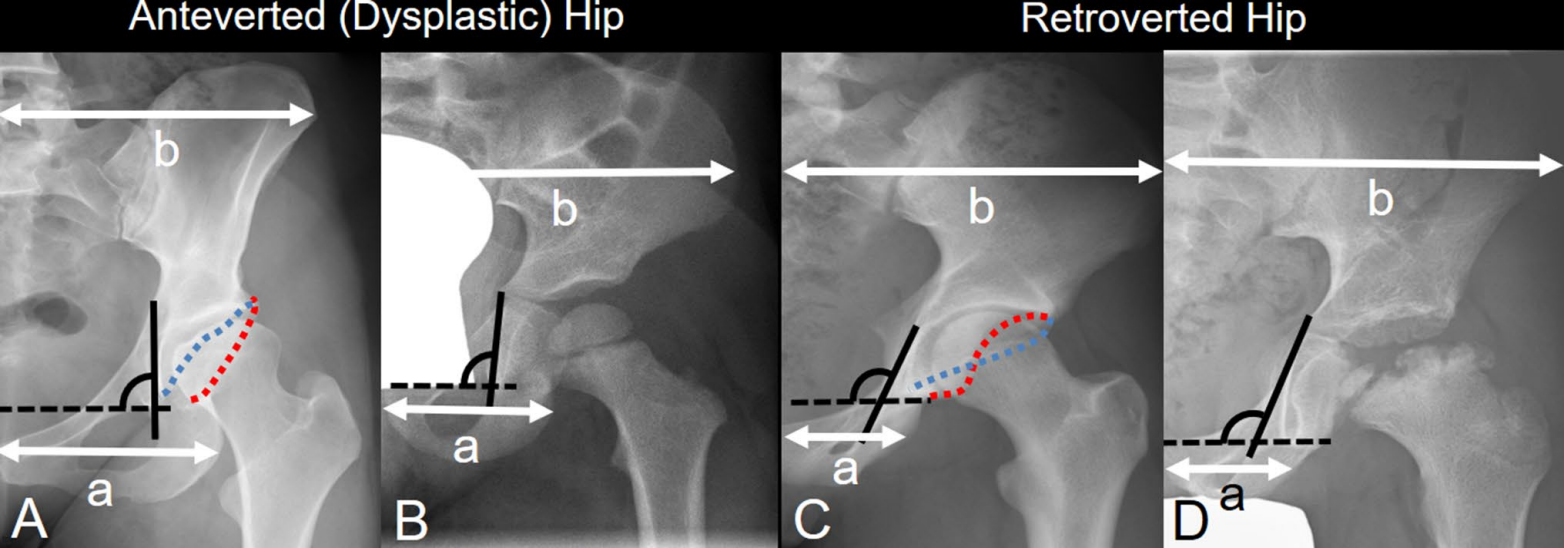
Acetabular version in **A** adults with an ossified acetabular morphology can be assessed by outlining the anterior (blue dotted line) and posterior (red dotted line) acetabular rim. **B** In children, the acetabular rims are not yet ossified and, therefore, acetabular version can not be assessed directly. **C** Acetabular retroversion in adults is recognized by the crossing of anterior and posterior acetabular wall (blue and red dotted lines). **D** In children with Legg–Calvé–Perthes disease (LCPD) and a non-ossified acetabulum the version can be evaluated using indirect parameters including the pelvic width index [22] (ratio of ‘a’ to ‘b’ in the upper figure) and the ilioischial angle (black angle) [22]. The smaller the pelvic width index or the higher the ilioischial angle, the more retroverted the acetabulum. For detailed description of these parameters see Table 2 and Fig. 2

Standard radiographic parameters to directly evaluate acetabular retroversion (Table 2) are the ischial spine sign [10] (Fig. 2A), the cross over sign [8, 16] (Fig. 2B), the retroversion index [16, 23] (Fig. 2C) and the posterior wall sign [16, 23] (Fig. 2D). These radiographic signs require an ossified acetabulum or ischial spine to be determined. Complete closure of the triradiate cartilage of the acetabulum typically occurs in female and male at the age of 15 and 16 years, respectively [15]. Since LCPD usually occurs before triradiate cartilage closure, the standard radiographic signs for acetabular retroversion cannot be assessed in children with LCPD. Therefore, we used two new and indirect radiographic parameters to determine acetabular version before ossification: the pelvic width index (Fig. 2E, Table 2) and the ilioischial angle (Fig. 2F, Table 2). These indirect radiographic parameters [22] allow evaluation of acetabular version in children with acute LCPD and a non-ossified anatomy of the acetabulum (Fig. 3). Both parameters have previously been validated and shown to have an excellent inter- and intraobserver reliability [22]. Acetabular version was evaluated on anteroposterior pelvic radiographs in all four stages according to Waldenström with the pelvic width index [22] and the ilioischial angle [22] and in addition with the four standard parameters in stage IV (healed stage; Fig. 1). Acetabular version was also compared to the contralateral and unaffected side. The mean radiographic follow-up was 7.0 ± 4.4 (2–23) years. Since no threshold for the pelvic width index and the ilioischial angle exist to define acetabular retroversion in children, a receiver operating characteristic (ROC) curve was calculated based on the retroversion index [21, 23] measured in hips in stage IV. The largest area under the ROC curve was found for a pelvic width index < 0.44 and an ilioischial angle > 97°. These thresholds were used to define acetabular retroversion in LCPD in children in all four Waldenström stages.

**Table 2:** Definitions of the radiographic parameters to assess acetabular retroversion (See Fig. 2 for illustration): common (direct) parameters to assess acetabular retroversion require an ossified acetabulum and include the ischial spine sign [10], cross-over sign [8, 16], retroversion index [16, 23] and posterior wall sign [16, 23]. Indirect radiographic parameters allow to assess acetabular retroversion in hips without an ossified acetabulum in children and include the pelvic width index [22] and the ilioischial angle [22]

Radiographic Parameter	Description
Direct parameters to assess acetabular retroversion (requiring an ossified acetabulum)	
Ischial spine sign [10] (Fig. 2A)	Positive if the ischial spine is projected medially to the pelvic brim
Cross-over sign [8, 16] (Fig. 2B)	Positive if the anterior acetabular rim crosses the course of the posterior acetabular rim
Retroversion index [16, 23] (Fig. 2C)	Allows to quantify the amount of acetabular retroversion. Ratio of ‘a’ to ‘b’ expressed as percentage. ‘a’ is defined as the distance of the cranial acetabular opening where the anterior rim is projected laterally to the posterior rim. ‘b’ is defined as entire width of the acetabular opening
Posterior wall sign [16, 23] (Fig. 2D)	Positive if the posterior acetabular wall runs medial to the femoral head center
Indirect parameters to assess acetabular retroversion (can be applied in hips without an ossified acetabulum)	
Pelvic width index [22] (Fig. 2E)	Ratio of width of ischium (a) to width of ileum (b). ‘a’ is defined from symphysis to the most lateral point of the ischial tuberosity. ‘b’ is defined from center of the sacrum to the most lateral point of the iliac wing. Both distances are measured parallel to the interteardrop line
Ilioischial angle [22] (Fig. 2F)	Angle between the interteardrop line and a line connecting the intersection of the iliopectineal and iliioschial lines and a second point on the most lateral aspect on the ipsilateral obturator foramen

To answer the first research question, (1) the pelvic width index, the ilioischial angle and the prevalence of acetabular retroversion (using the thresholds for the pelvic width index and ilioischial angle) were compared among the Waldenström stages. For the second question, the pelvic width index, the ilioischial angle and the prevalence of acetabular retroversion were compared among the Waldenström stages for the contralateral unaffected side and compared to the LCPD side. The third question regarding possible factors predictive for acetabular retroversion in Waldenström stage II or IV, we tested several demographic and radiographic parameters. This included age at different stages of Waldenström, gender, subluxation of the femoral head (disrupted Shenton line [9]), collapse of the lateral pillar in the fragmentation stage (Herring lateral pillar classification [7]), dysplastic shape of the acetabulum (LCE angle < 20°) [14, 25] in stage IV or sphericity of the femoral head and congruency of the joint in stage IV (Stulberg classification [18]).

Normal distribution was confirmed using the Kolmogorov–Smirnov test. We compared the pelvic width index and the ilioischial angle among the four stages of Waldenström using the multivariate analysis of variance (MANOVA). If differences existed, pairwise comparison was performed with the paired *t* test. The pelvic width index and the ilioischial angle were compared between the hip with LCPD and the unaffected contralateral hip using the unpaired *t* test. Predictive factors for acetabular retroversion were calculated using logistic regression analysis. All statistical analysis was performed using SPSS (Version 25, SPSS IBM Statistics, Armonk, New York, USA).

## Results

**Fig. 4:**
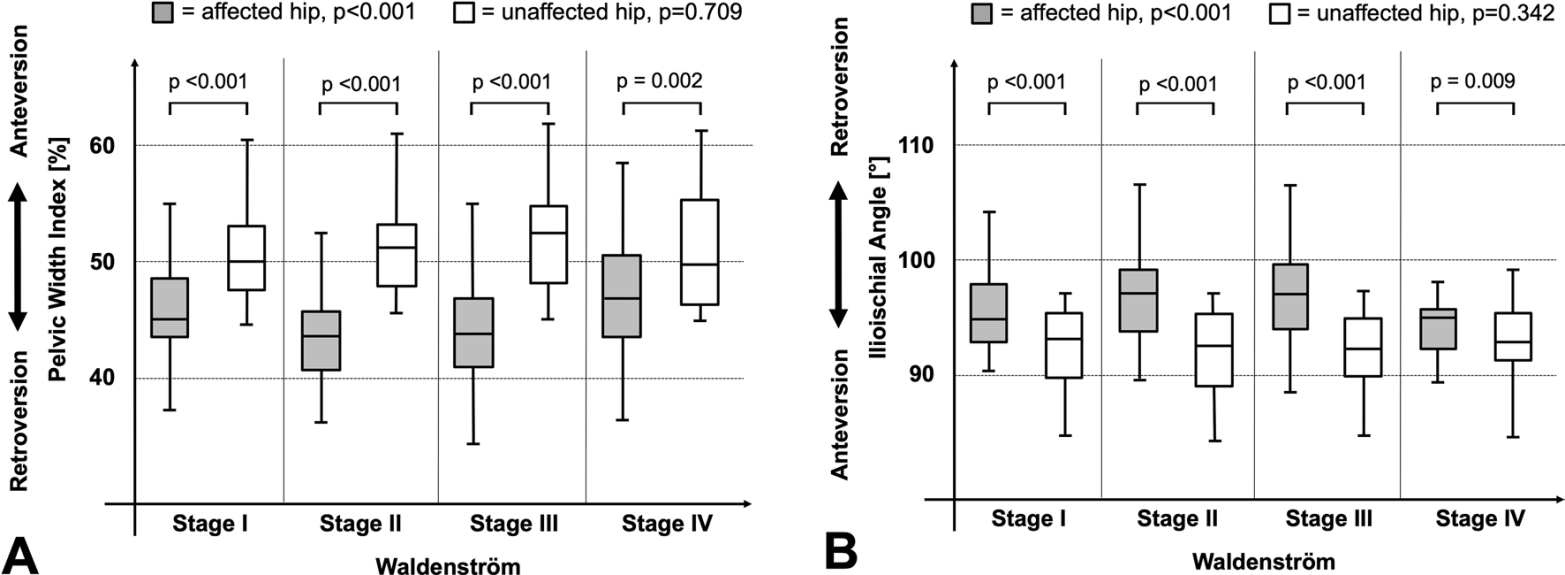
Results for acetabular version in hips with Legg–Calvé–Perthes Disease (LCPD) according to the Waldenström stages [24]. Compared to the non-affected hip, hips with LCPD showed **A** a decreased pelvic with index [22] in all four Waldenström stages [24] (*p* values < 0.002) and **B** an increased ilioischial angle [22] in all four Waldenström stages (*p *values < 0.009)

**Fig. 5:**
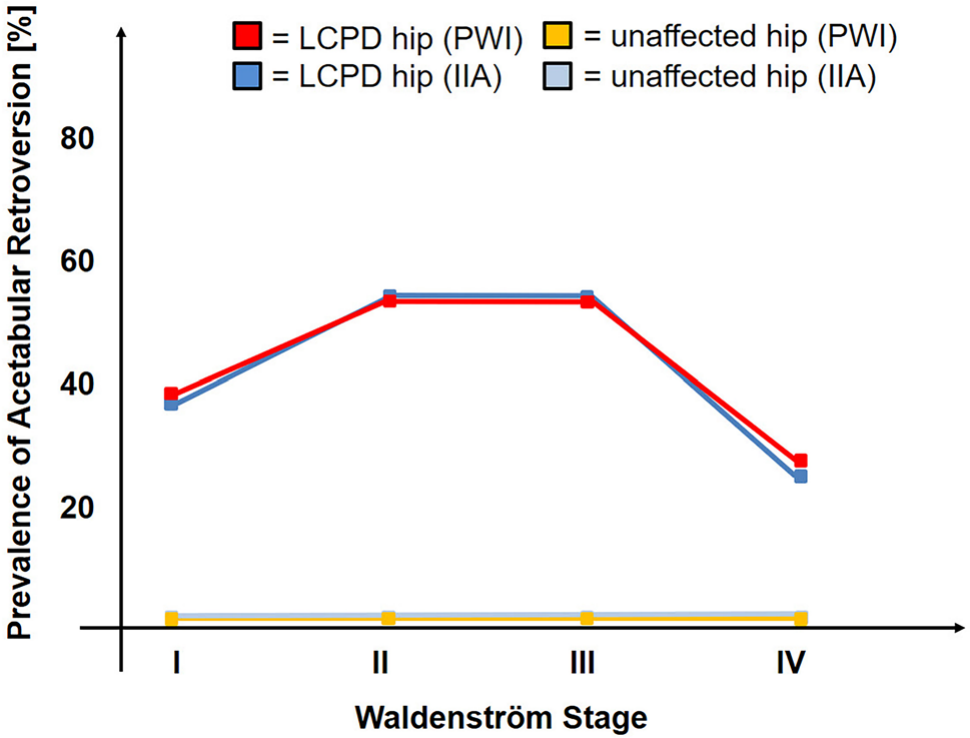
The prevalence of acetabular retroversion during the course of the Legg–Calvé–Perthes disease (LCPD) was increased compared to the unaffected, contralateral hip (*p* values < 0.010), showed the highest prevalence in Waldenström [24] stages 2 and 3 and decreased in stage 4 with healed LCPD (*p* < 0.013 for the pelvic width index [22] and *p* < 0.001 for the ilioischial angle [22]; see Table 3 for detailed information of *p* values). For both indirect parameters to assess acetabular retroversion (pelvic width index [22] and ilioischial angle [22]) comparable results regarding prevalence of acetabular retroversion were found

The pelvic width index (*p* < 0.001) and the ilioischial angle (*p* < 0.001) differed significantly among the four stages of Waldenström (Fig. 4A and B). The mean pelvic width index decreased to 44% in stage II and III (Table 3; *p* < 0.001). The mean ilioischial angle increased to a maximum of 98° and 97° in stage II and III (Table 3; *p* < 0.001). Both parameters indicated a more retroverted acetabulum in stage II and III. The prevalence of acetabular retroversion differed among the four Waldenström stages for the pelvic width index (*p* = 0.038) and ilioischial angle (*p* = 0.003; Table 3). The highest prevalence of retroversion was found in stage II and III for both parameters ranging from 54 to 56% compared to stage I and IV with a prevalence ranging from 23 to 39% (Table 3 and Fig. 5).

**Table 3:** Radiographic results for acetabular version in hips with Legg–Calvé–Perthes Disease (LCPD) staged according to Waldenström [24] (55 hips) and compared to the contralateral hip without LCPD (47 hips)

Parameter	Waldenström stage I (41 hips)	Waldenström stage II (54 hips)	Waldenström stage III (52 hips)	Waldenström stage IV (43 hips)	*p *value global	*p* value stage I vs. II	*p* value stage II vs. III	*p* value stage III vs. IV
Pelvic width index (%)								
LCPD	46 ± 4 (37–55)	44 ± 5 (32–56)	44 ± 5 (34–58)	48 ± 5 (36–62)	< 0.001	< 0.001	0.621	< 0.001
Contralateral side without LCPD	51 ± 4 (45–62)	52 ± 4 (45–65)	52 ± 4 (45–63)	51 ± 5 (45–2)	0.709	–	–	–
*p* value LCPD vs. contralateral	< 0.001	< 0.001	< 0.001	0.002				
Ilioischial angle (°)								
LCPD	96 ± 4 (90–106)	98 ± 4 (89–110)	97 ± 4 (88–109)	94 ± 4 (84–109)	< 0.001	< 0.001	0.201	< 0.001
Contralateral side without LCPD	92 ± 4 (85–97)	92 ± 4 (84–97)	92 ± 4 (79–97)	92 ± 3 (82–98)	0.342	–	–	–
*p* value LCPD vs. contralateral	< 0.001	< 0.001	< 0.001	0.009				
Prevalence of acetabular retroversion (hips [percentage])								
Pelvic width Index (PWI)								
LCPD (defined as PWI < 0.44%)	16 (39)	29 (54)	29 (56)	13 (30)	0.038	0.156	0.831	0.013
Contralateral side without LCPD	0	0	0	0	1.000	–	–	–
*p *value LCPD vs. contralateral	< 0.001	< 0.001	< 0.001	< 0.001				
Ilioischial angle (IIA)								
LCPD (defined as IIA > 97°)	15 (37)	30 (56)	29 (56)	10 (23)0	0.003	0.067	0.982-	0.001
Contralateral side without LCPD	0	0	0	0	1.000	–	–	–
*p* value LCPD vs. contralateral	< 0.001	< 0.001	< 0.001	0.001				

Continuous parameters are expressed as mean ± standard deviation and range in parenthesis

*LCPD* Legg-Calvé-Perthes Disease

In hips of the contralateral side without LCPD the pelvic width index (*p* = 0.709) and the ilioischial angle (*p* = 0.342) did not differ among the four stages of Waldenström (Table 3). Compared to the LCPD side, the pelvic width index was increased (Fig. 4A, *p* < 0.002) and the ilioischial angle was decreased (Fig. 4B, *p* < 0.009) for the contralateral side in all Waldenström stages.

The prevalence of acetabular retroversion in hips without LCPD was 0% in all stages and for both pelvic width index and ilioischial angle (Table 3).

There were three predictive factors for acetabular retroversion in LCPD (Table 4): younger age, a non-dysplastic shape of the acetabulum and a collapse of the lateral pillar in stage II. Age less than 6 years in Waldenström stage II had a 2.4 (range 1.3–4.2, *p* = 0.004) hazard ratio (HR) to develop acetabular retroversion in stage II and 4.2 (1.1–16.8, *p* = 0.042) HR to develop retroversion in stage IV (Table 4). Non-dysplastic hips (lateral center edge [LCE] angle > 20° [14, 25]) in stage IV had an increased risk to develop acetabular retroversion in stage IV (HR 5.0 [1.7–10.0], *p* = 0.008, Table 4). Hips with a beginning or advanced collapse of the lateral pillar in stage II (Herring lateral pillar classification group B or C) had a HR of 3.6 (1.1–12.0, *p* = 0.036) to develop retroversion in stage IV (Table 4).

**Table 4:** Predictive factors for acetabular retroversion in stages II or IV according to Waldenström [24] in hips with Legg–Calvé–Perthes disease

	Parameter	Hazard ratio (95% confidence interval)	*p* value
Retroversion in Stage II	Age at stage II < 6 years	2.4 (1.3–4.2)	0.004
	Male gender	0.8 (0.2–3.1)	0.372
	Shenton line [9] not intact at stage II	0.5 (0.2–1.5)	0.220
	Herring lateral pillar classification [7] B or C at stage II*	2.5 (0.9–6.4)	0.066
Retroversion in Stage IV	Age at stage II < 6 years	4.2 (1.1–16.8)	0.042
	Age at stage IV < 12 years	1.4 (1.1–1.7)	0.007
	Male gender	2.3 (0.4–12.6)	0.342
	Non-dysplastic acetabulum (LCE angle < 20°) at stage IV	5.0 (1.7–10.0)	0.008
	Shenton line [9] not intact at stage IV	1 (0.7–3.3)	0.245
	Herring lateral pillar classification [7] group B or C at stage II*	3.6 (1.1–12.0)	0.036
	Stulberg classification [18] ≥ 3 at stage IV^†^	2.5 (0.6–5)	0.183

*hips with loss of height of the lateral pillar (Herring lateral pillar classification group B and C) were compared to those without of loss of height (Group A); ^†^hips with a spherical head (Stulberg Class 1 or 2) were compared to hips with an aspherical or flat femoral head (Class 3 to 5)

## Discussion

**Fig. 6:**
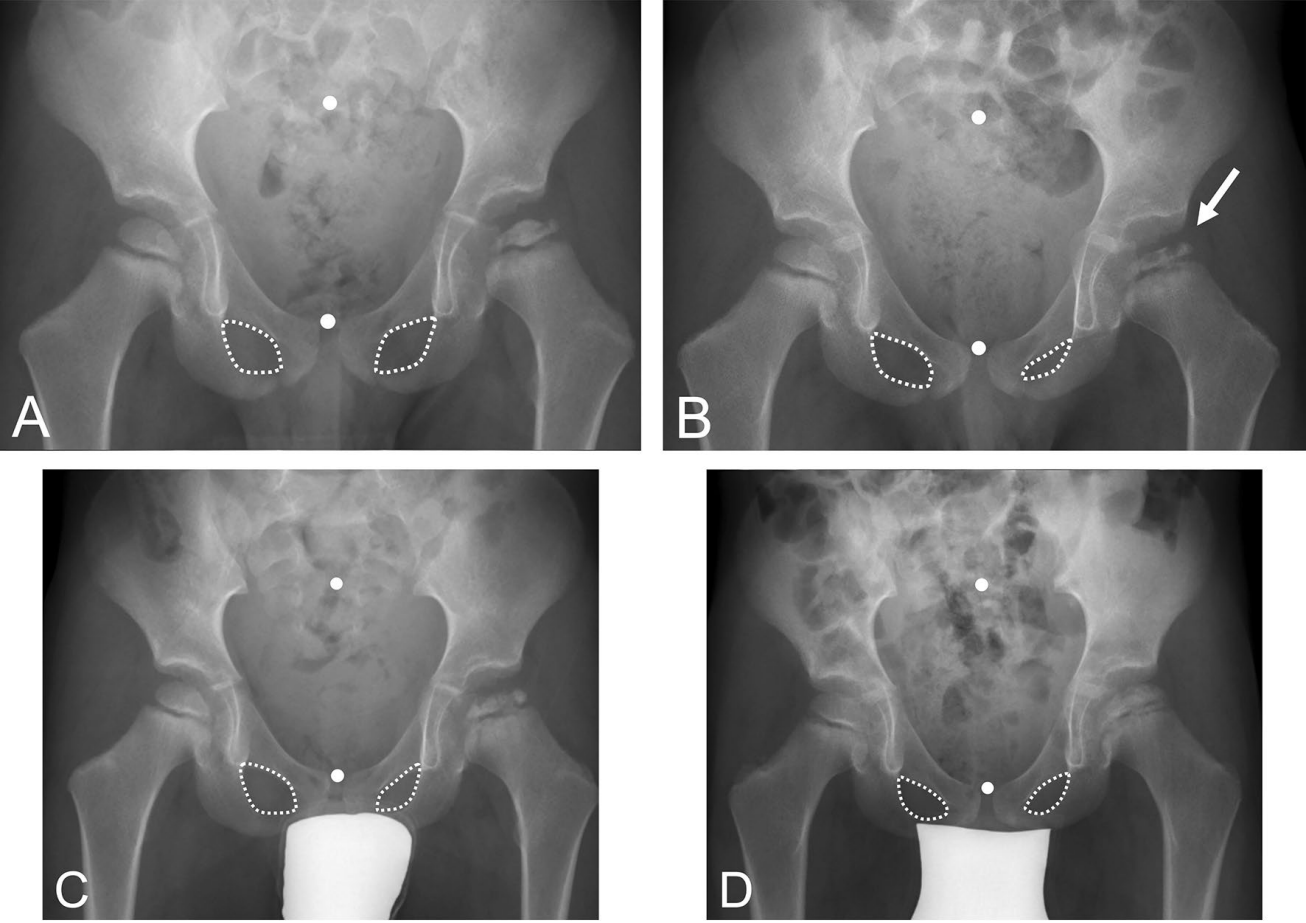
Four-year old boy with Legg–Calvé–Perthes Disease (LCPD) on the left hip at **A** the initial stage (Waldenström I [24]) showing a symmetrical shape of the obturator foramen. **B** At the fragmentation stage (arrow, Waldenström II [24]), an asymmetrical shape of the obturator foramen indicating acetabular retroversion can be seen despite correct pelvic position during radiograph acquisition. **C** With progressive healing of LCPD the foramen become more symmetrical again (Waldenström III [24]) but **D** some asymmetry remains in the healed stage (Waldenström IV [24]). The white dots (Fig. 6A–D) indicate the mid of the sacrum and symphysis indicating correct pelvic rotation and constant pelvic tilt during radiograph acquisition

Among various disorders of the hip, LCPD has been shown to have the highest prevalence of acetabular retroversion ranging up to 31–49% [3, 5, 11]. Retroversion of the acetabulum further restricts range of motion and aggravates femoroacetabular impingement in hips with LCPD [20]. We observed that the prevalence of acetabular retroversion is increased in hips with unilateral LCPD compared to the contralateral, unaffected side (Fig. 4). In addition, the prevalence increases in the fragmentation and early bone formation stages (Waldenström stage II and III) in hips with LCPD (Fig. 5). In some of the hips with acetabular retroversion, normal version was observed in the healing stage (Waldenström stage IV; Fig. 6A–D). The contralateral side without LCPD rarely showed changes in acetabular version over the same time period (Fig. 5). Younger children, and those with advanced femoral head collapse were at higher risk to develop retroversion (Table 4). In contrast, a dysplastic shaped acetabulum was associated with a decreased risk of retroversion (Table 4).

There are limited reports and contradictory findings about the prevalence of acetabular retroversion in skeletally immature hips with LCPD (Table 5). We found a prevalence of 37–39% in the very early stage of LCPD (Waldenström stage I) and the highest prevalence of 54–56% in stages II or III with fragmentation and new bone formation (Fig. 5). Larson et al. [12] found a substantially higher prevalence of acetabular retroversion of 90% in skeletally immature hips with LCPD when using the ischial spine sign for evaluation. In our experience, the ischial spine sign can be difficult to evaluate in skeletally immature hips since the ischial spine is not always visible due to the lack of ossification. Sankar et al. [17] have evaluated acetabular version in skeletally immature hips with LCPD using CT and MRI images. They defined acetabular retroversion on axial images at the level of the femoral head center with an angle of the acetabular opening being oriented posteriorly [17]. Using this definition, a prevalence of only 2% of retroversion in LCPD was found [17]. This is significantly lower than the prevalence of retroversion in the current study or any literature about this topic (Table 5) which might be due to the strict definition of acetabular retroversion in CT and MRI images. On conventional radiographs, retroversion appears earlier due to the conical projection of x rays compared to the parallelism of rays in CT or MRI. In skeletally mature hips and healed LCPD, the prevalence of acetabular retroversion ranges from 31 to 49% in literature [3, 5, 11, 17]. We found comparable results with a prevalence of 23–30% using the indirect radiographic parameters (Table 3) and 30–39% with the standard radiographic parameters (Table 1).

**Table 5:** Selected literature about the acetabular version in hips of children with Legg–Calvé–Perthes disease (LCPD)

Author (year)	Study setup	Number of patients (hips)	Mean age and follow-up (years)	Results
Sankar and Flynn [17] (2008)	Evaluation of version angle on axial CT or MRI slices in skeletally immature patients. Follow-up past skeletal maturity in 36% of patients with evaluation of cross-over sign on radiographs	44 (53)	7 (2–12) n.a.	No difference in acetabular version on axial slices between LCPD (average version of 15°, range -5°–24°) compared to contralateral hip without LCPD. Following skeletal maturity, 31% of hips with LCPD showed a positive cross-over sign. Herring lateral pillar classification [7] (hips in Group C with > 50% loss of height) was predictive for acetabular retroversion
Larson et al. [12] (2011)	Acetabular retroversion assessed on earliest radiograph available using ischial spine sign. Comparison to control group including 25 patients and 50 hips (trauma cases)	47 (49)	7 (4–12) n.a	Positive ischial spine sign in 90% of LCPD with open triradiate physis, 75% in LCPD with closed physis, 64% on contralateral side of unilateral LCPD and 32% in control group. No predictive factors
Yoshida et al. [26] (2016)	Evaluation of version angle on axial MRI at mean age of 7 (range 4–11) years and after a mean follow-up of 8 (3–14) years	25 (25)	7 (4–11) 8 (3–14)	No difference in anteversion for hips with LCPD between initial and follow-up status. At follow-up, hips with a round femoral head and LCPD (Stulberg [18] class I and II) were more retroverted compared to the contralateral hip. In hips with LCPD and an ovoid or flat head (Stulberg classification [18] class III and IV) no difference existed compared to the contralateral side at follow-up. No predictive factors
Liao et al. [13] (2021)	Acetabular version angle assessed on axial views of CT scans in 33 male and 7 female patients with unilateral LCPD. 95% of hips in Waldenström stage I or II	40 (40)	8 ± 2 n.a	Acetabular anteversion angle was minimally decreased (resembling more retroversion) on the affected side (10.6° ± 8.1°–12.5°) compared to the unaffected side (12.0° ± 9.0° – 13.3°; p = 0.002)
Current study	Repeated evaluation of acetabular version in skeletally immature and mature LCPD patients (Waldenström [24] stages I–IV) using pelvic width index, ilioischial angle and obturator index	51 (55)	6 (2–13) 7 (2–23)	Decreasing pelvic width and obturator indices and increasing ilioischial angle in the fragmentation and early bon formation stages (Waldenström [24] stages II and III) all indicating acetabular retroversion. Retroversion was associated with younger age, non-dysplastic shape a collapse of the lateral pillar in fragmentation stage

*n.a.* not applicable

We discovered a prevalence of acetabular retroversion of 0% in all stages of Waldenström for the contralateral and unaffected hip (Figs. 4 and 5). Compared to the LCPD hip, a more anteverted acetabulum was found for the contralateral unaffected hip in most studies (Larson et al. [12], Yoshida et al. [26], Liao et al. [13]). In only one study by Sankar et al. [17] no difference in acetabular version was observed, which might be due to difference in the imaging modality, definition of acetabular retroversion or stages of LCPD. Larson et al. [12] measured acetabular retroversion using the ischial spine sign and found in skeletally mature hips an increased prevalence of 75% in hips with LCPD compared to the unaffected contralateral hip with 64%, which in turn was higher than the prevalence of 32% found in a control group without LCPD in any hips.

In the current study, three parameters were predictive for acetabular retroversion in LCPD including younger age, head collapse (Herring lateral pillar classification B and C) and a non-dysplastic morphology of the acetabulum (Table 4). Previously, Sankar et al. [17] could also show that hips with advanced loss of height of the lateral pillar of > 50% (Group C according to the Herring lateral pillar classification) had an increased risk to develop acetabular retroversion. In literature, age or a non-dysplastic shape was not shown to be predictive for acetabular retroversion. We found a 4.2 times increased risk of acetabular retroversion in healed LCPD for children younger than 6 years at the fragmentation stage (Waldenström stage II; Table 4). For children younger than 12 years at the healed stage (Waldenström stage IV), the risk was 1.4 times increased for retroversion (Table 4). We speculate that the deformity of the femoral head in LCPD results in altered growth with retroversion of the acetabulum in skeletally immature hips. Our results suggest that the younger the children with LCPD, the more retroverted the acetabulum becomes, which could be due to increased plasticity of the bone. A more severe deformity (head collapse with Herring lateral pillar classification group B or C) of the femoral head would further increase the retroversion of the acetabulum. Eijer et al. [4] have previously speculated that in hips with LCPD, the primary deformity is the retroverted acetabulum which subsequently results in altered loading patterns of the joint and stress on the blood supply of the femoral head with secondary deformity of the femoral head [4]. Larson et al. [12] speculated about microtrauma to the retinacular vessels due to femoroacetabular impingement at the location of the vessels due to acetabular retroversion. The trauma to the retinacular vessels would eventually result in impaired femoral head perfusion resulting in LCPD [12]. Our findings are contradictory to these speculations since we could show that acetabular retroversion is present in only up to 39% after changes of the femoral head are observable. In addition, we could show that acetabular retroversion evolves during the course of the disease and partially resolves in the healing stage.

The study has several limitations. First, the study comprises a selected series of children with exclusion of children with LCPD but no radiographs for at least three out of four Waldenström stages (exclusion rate of 44%). This strict inclusion criterion minimizes the effect of missing radiographs on the measured acetabular version due to individual differences in version rather than changes over time. Second, the pelvic orientation during radiograph acquisition has a direct implication on the radiographic anatomy of the pelvis. Therefore, we excluded all radiographs from evaluation without correct centering or malorientation of the pelvis. Tilt also directly affects acetabular version on radiographs, however, both hips to the same extent. Since, the non-affected side did not show any changes of acetabular orientation over time, we assume that pelvic tilt might not have substantially affected the measurements. Third, no normal cut-off values for acetabular retroversion for the pelvic width index or ilioischial angle in children exist. The parameters have been validated by comparing dysplastic and retroverted hips in adults [22]. Therefore, we defined the threshold for acetabular retroversion for both of these radiographic parameters in skeletally mature hips in Waldenström stage IV by comparing them to the retroversion index with a ROC analysis. With this method, we found a comparable prevalence of acetabular retroversion (Tables 1 and 3) using the direct and indirect parameters (Table 2).

## Conclusion

Understanding of the altered pelvic morphology in hips with acetabular retroversion permits the evaluation of acetabular version even in skeletally immature hips prior to complete ossification. To the best of the authors’ knowledge, the current study is the first study evaluating acetabular version in children with LCPD from early stage to healing. In comparison with the unaffected, contralateral hip, we could show that acetabular retroversion is associated with LCPD in up to 56%. However, acetabular retroversion is not a constant deformity over the course of the disease and a potential of correction of acetabular version exists in some hips with healed LCPD (Fig. 6). This has a potential clinical impact on the timing and type of surgical correction, especially in pelvic osteotomies for correction of acetabular version. These interventions are typically performed in the fragmentation stage due to the highest capacity of remodeling, the stage where we found the highest prevalence of acetabular retroversion. The clinical impact of these findings on surgical interventions needs further evaluation.

## Data Availability

All data generated or analyzed during the study are included in this article. No materials were sampled for this study.

## References

[1] Albinana J, Morcuende JA, Delgado E, Weinstein SL (1995). Radiologic pelvic asymmetry in unilateral late-diagnosed developmental dysplasia of the hip. J Pediatr Orthop.

[2] Barker DJ, Hall AJ (1986). The epidemiology of Perthes' disease. Clin Orthopaed Relat Res.

[3] Berg RP, Galantay R, Eijer H (2010). Retroversion of the contralateral adult acetabulum after previous Perthes' disease. Acta Orthop Belg.

[4] Eijer H (2007). Towards a better understanding of the aetiology of Legg-Calve-Perthes' disease: acetabular retroversion may cause abnormal loading of dorsal femoral head-neck junction with restricted blood supply to the femoral epiphysis. Med Hypotheses.

[5] Ezoe M, Naito M, Inoue T (2006). The prevalence of acetabular retroversion among various disorders of the hip. J Bone Jt Surg.

[6] Fujii M, Nakashima Y, Sato T, Akiyama M, Iwamoto Y (2011). Pelvic deformity influences acetabular version and coverage in hip dysplasia. Clin Orthop Relat Res.

[7] Herring JA, Neustadt JB, Williams JJ, Early JS, Browne RH (1992). The lateral pillar classification of Legg-Calve-Perthes disease. J Pediatr Orthop.

[8] Jamali AA, Mladenov K, Meyer DC (2007). Anteroposterior pelvic radiographs to assess acetabular retroversion: high validity of the "cross-over-sign". J Orthopaed Res Off Publ Orthopaed Res Soc.

[9] Jones DH (2010). Shenton's line. J Bone Jt Surg Br.

[10] Kalberer F, Sierra RJ, Madan SS, Ganz R, Leunig M (2008). Ischial spine projection into the pelvis: a new sign for acetabular retroversion. Clin Orthop Relat Res.

[11] Kawahara S, Nakashima Y, Oketani H (2012). High prevalence of acetabular retroversion in both affected and unaffected hips after Legg-Calve-Perthes disease. J Orthopaed Sci Off J Jap Orthopaed Assoc.

[12] Larson AN, Stans AA, Sierra RJ (2011). Ischial spine sign reveals acetabular retroversion in Legg-Calve-Perthes disease. Clin Orthop Relat Res.

[13] Liao S, Zhao M, Wang T (2021). Retroversion of the hemipelvis rather than hypoplastic posterior wall decreases acetabular anteversion in hips affected by Perthes disease. Sci Rep.

[14] Murphy SB, Ganz R, Muller ME (1995). The prognosis in untreated dysplasia of the hip. A study of radiographic factors that predict the outcome. J Bone Jt Surg.

[15] Parvaresh KC, Pennock AT, Bomar JD, Wenger DR, Upasani VV (2018). Analysis of acetabular ossification from the triradiate cartilage and secondary centers. J Pediatr Orthop.

[16] Reynolds D, Lucas J, Klaue K (1999). Retroversion of the acetabulum. A cause of hip pain. J Bone Jt Surg Br.

[17] Sankar WN, Flynn JM (2008). The development of acetabular retroversion in children with Legg-Calve-Perthes disease. J Pediatr Orthop.

[18] Stulberg SD, Cooperman DR, Wallensten R (1981). The natural history of Legg-Calve-Perthes disease. J Bone Jt Surg.

[19] Suzuki S (1995). Deformity of the pelvis in developmental dysplasia of the hip: three-dimensional evaluation by means of magnetic resonance image. J Pediatr Orthop.

[20] Tannast M, Hanke M, Ecker TM, Murphy SB, Albers CE, Puls M (2012). LCPD: reduced range of motion resulting from extra- and intraarticular impingement. Clin Orthop Relat Res.

[21] Tannast M, Hanke MS, Zheng G, Steppacher SD, Siebenrock KA (2015). What are the radiographic reference values for acetabular under- and overcoverage?. Clin Orthop Relat Res.

[22] Tannast M, Pfannebecker P, Schwab JM, Albers CE, Siebenrock KA, Buchler L (2012). Pelvic morphology differs in rotation and obliquity between developmental dysplasia of the hip and retroversion. Clin Orthop Relat Res.

[23] Tannast M, Siebenrock KA, Anderson SE (2007). Femoroacetabular impingement: radiographic diagnosis–what the radiologist should know. AJR Am J Roentgenol.

[24] Waldenstrom H (1984). The classic. The first stages of coxa plana by Henning Waldenstrom. Clin Orthopaed Relat Res.

[25] Werner CM, Ramseier LE, Ruckstuhl T (2012). Normal values of Wiberg's lateral center-edge angle and Lequesne's acetabular index–a coxometric update. Skeletal Radiol.

[26] Yoshida T, Kim WC, Nishida A (2016). Acetabular anteversion angle from early stage of Perthes disease to adolescence. J Orthop.

